# Ecological and Reproductive Cycles Drive Henipavirus Seroprevalence in the African Straw‐Coloured Fruit Bat ( *Eidolon helvum* )

**DOI:** 10.1002/ece3.70555

**Published:** 2024-11-11

**Authors:** Maya M. Juman, Louise Gibson, Richard D. Suu‐Ire, Sylvester Languon, Osbourne Quaye, Grace Fleischer, Samuel Asumah, E. Rosa Jolma, Avinita Gautam, Spencer L. Sterling, Lianying Yan, Christopher C. Broder, Eric D. Laing, James L. N. Wood, Andrew A. Cunningham, Olivier Restif

**Affiliations:** ^1^ Department of Veterinary Medicine University of Cambridge Cambridge UK; ^2^ Institute of Zoology Zoological Society of London London UK; ^3^ School of Veterinary Medicine, College of Basic and Applied Sciences University of Ghana Accra Ghana; ^4^ West African Centre for Cell Biology of Infectious Pathogens, Department of Biochemistry, Cell and Molecular Biology, College of Basic and Applied Sciences University of Ghana Accra Ghana; ^5^ Wildlife Division of Forestry Commission Accra Ghana; ^6^ Department of Coastal Systems NIOZ Royal Netherlands Institute for Sea Research Yerseke The Netherlands; ^7^ Department of Population Health Sciences, Veterinary Medicine Utrecht University Utrecht The Netherlands; ^8^ DST‐CIMS, Institute of Science Banaras Hindu University Varanasi India; ^9^ Uniformed Services University of the Health Sciences Bethesda Maryland USA; ^10^ Henry M. Jackson Foundation for the Advancement of Military Medicine Bethesda Maryland USA

**Keywords:** *Eidolon helvum*, Ghana, henipaviruses, life history, multiplex, paramyxoviruses, reproductive ecology, serology

## Abstract

Bats are known to host zoonotic viruses, including henipaviruses that cause high fatality rates in humans (Nipah virus and Hendra virus). However, the determinants of zoonotic spillover are generally unknown, as the ecological and demographic drivers of viral circulation in bats are difficult to ascertain without longitudinal data. Here we analyse serological data collected from African straw‐coloured fruit bats ( *Eidolon helvum* ) in Ghana over the course of 2 years and across four sites, comprising three wild roosts and one captive colony. We focus on antibody affinity to five henipavirus antigens: Ghanaian bat henipavirus (GhV), Nipah virus (NiV), Hendra virus (HeV), Mojiang virus (MojV) and Cedar virus (CedV). In the wild roosts, we detected seasonal variations in henipavirus antibody binding, possibly associated with bat life‐history cycles and migration patterns. In the captive colony, we identified increases in antibody affinity levels among pregnant bats, suggesting possible shifts in the immune system during pregnancy. These bats then pass maternal antibodies to their pups, which wane before antibody affinity levels rise later in life following initial infections and/or reactivation of latent infections. These results improve our understanding of the links between bat ecology and viral circulation, including for GhV, a locally‐circulating African henipavirus.

## Graphical abstract

Host ecology is critical to understanding the circulation and maintenance of viruses in host populations. We identify seasonal variations in henipavirus seroprevalence in Ghanaian straw‐coloured fruit bats, possibly associated with bat life‐history cycles and migration patterns. These results improve our understanding of the links between bat ecology and viral circulation, including for Kumasi virus, a locally‐circulating African henipavirus.

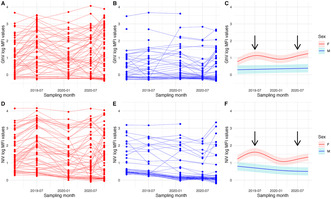

## 1 Introduction

Bats are recognised as important hosts of zoonotic pathogens (Calisher et al. 2006). Various bat species have either been identified or suspected as hosts of many emerging viruses (Letko et al. 2020), possibly due to the unique chiropteran immune system (Irving et al. 2021) or the high species richness of this order (Mollentze and Streicker 2020). The widespread hunting of bats for meat as well as the growing presence of bats in urban areas create opportunities for contact with humans and subsequent viral spillover (Letko et al. 2020). Despite the attention bat‐borne viruses have received in recent years, our understanding of how these viruses persist and circulate within host populations is lacking. Such knowledge is critical for predicting and mitigating zoonotic spillover risk.

Ecological and life history patterns are hypothesised to drive transmission dynamics within bat populations and downstream spillover risk (Plowright et al. 2017). For example, seasonality in bat‐virus prevalence and shedding has been identified in different systems (Plowright et al. 2011; Amman et al. 2012; Field et al. 2015; Dietrich et al. 2015; Joffrin et al. 2020; Mortlock et al. 2021; Montecino‐Latorre et al. 2022; Becker et al. 2023). In Australia, reproductive cycles, nutritional stress and age‐related factors have been linked to higher rates of Hendra virus detection in several *Pteropus* species (Plowright et al. 2008; Giles et al. 2018; Edson et al. 2019). Serology offers a proxy for past or current infection in these systems. Together with molecular detection of viral RNA, it allows for an in‐depth examination of the history of viral circulation in a population (Hayman et al. 2008, 2010, 2012; Peel et al. 2013). Longitudinal and cross‐sectional serological surveys also enable the mechanistic modelling of transmission within populations, although antibody cross‐reactivity can limit the specificity of these findings for particular viruses (Peel et al. 2018; Brook et al. 2019; Glennon et al. 2019).

One particular host species of interest is *Eidolon helvum* , the African straw‐coloured fruit bat, a migratory bat widely distributed throughout sub‐Saharan Africa and several small offshore islands (DeFrees and Wilson 1988). A growing body of evidence suggests that *E. helvum* hosts many potentially zoonotic viruses, including henipaviruses, a genus of paramyxovirus. Prototypic henipaviruses, Hendra (HeV) and Nipah (NiV) viruses, cause high fatality rates in infected humans (Eaton et al. 2006). Several studies have found henipavirus antibodies in *E. helvum* populations across the entire African continent and on islands in the Gulf of Guinea (Hayman et al. 2008; Peel et al. 2013; Cantoni et al. 2023). African henipavirus RNA was first detected in 2008 in faeces from an urban *E. helvum* roost in Kumasi, Ghana; a novel virus—Ghanaian bat henipavirus (GhV)—was sequenced and found to be phylogenetically similar to HeV and NiV (Drexler et al. 2009, 2012). RNA from other African bat henipaviruses and “henipa‐like” paramyxoviruses has since been detected in both wild and captive *E. helvum* (Drexler et al. 2012; Gibson et al. 2021; Jolma et al. 2021). The zoonotic potential of GhV and other African henipaviruses remains unknown (Pernet et al. 2014), but as the first henipaviruses identified outside of Asia and Australia, they should be monitored and investigated within a One Health framework.

Repeated sampling of individual bats elucidates viral antibody dynamics in populations, but wild *E. helvum* live in very large colonies and are rarely recaptured. Captive colonies of such colonial species provide a unique opportunity for longitudinal monitoring, albeit under artificial ecological conditions. To address the limitations of both of these methods, we designed a study that includes longitudinal sampling across 2 years from three wild roosts and a captive colony of *E. helvum* in Ghana. The captive colony was established in Greater Accra in 2010 with 77 bats caught locally. Since then, these bats have been breeding with no direct or indirect external bat contact, maintaining a stable population size of around 150–200 individuals of known ages. Earlier studies of antibody dynamics in this population indicated that these bats experience persistent or recurring henipavirus infection (Baker et al. 2014; Glennon et al. 2019). Recent follow‐up studies have revealed that despite the small size of the captive colony and its isolation from wild bats, multiple paramyxoviruses continue to circulate within the closed population, with seasonal RNA shedding patterns varying between specific viruses (Gibson et al. 2021; Jolma et al. 2021).

Here we analyse the serological dynamics in the captive colony over a 2‐year period after nearly a decade of isolation and compare them to antibody levels in three wild roosts in southern Ghana. Although variations in antibody levels only provide an indirect and incomplete proxy for infection dynamics with unknown numbers of viruses, direct detection of henipavirus RNA in individual bats (e.g., in urine samples) remains very difficult due to low copy numbers. We tested bat serum samples against a panel of antigens from six paramyxoviruses using a binding assay: GhV, NiV, HeV, Mojiang virus (MojV), Cedar virus (CedV) and Menangle virus (MenV). The first five of these are classified as henipaviruses (Drexler et al. 2009), while MenV belongs to the *Pararubulavirus* genus. Despite a growing amount of data on the genetic diversity of bat paramyxoviruses (Drexler et al. 2009; Mortlock et al. 2015; Letko et al. 2020), little is known about their antigenic diversity apart from the strong cross‐reactivity between Hendra and Nipah G proteins (Ruhs et al. 2023). While constrained by the availability of purified viral antigens, our diverse panel was assembled with the aim of detecting exposure to (or infection with) a broader set of paramyxoviruses than in earlier studies. In addition to GhV, paramyxoviruses similar to NiV and HeV are present in wild *E. helvum* in Ghana as well as in the captive colony (Drexler et al. 2009, 2012; Gibson et al. 2021; Jolma et al. 2021). However, due to reagent availability, most serosurveys of paramyxoviruses in African bats to date have used NiV or HeV antigens. The serological dynamics of GhV in *E. helvum* have never been examined before, despite some evidence of African henipaviruses spilling over into human populations in nearby Cameroon (Pernet et al. 2014). Hence, we were particularly interested in comparing serological signals from the NiV and GhV antigens.

This study is the first to compare antibody levels against paramyxoviruses between wild and captive bats in Africa over several months using a panel of six antigens. Using this dataset, we sought to investigate the ecological drivers of bat‐virus infection dynamics. Specifically, we analysed seasonal and/or spatial patterns in wild roosts as well as demographic factors and reproductive cycles in the captive colony.

## 2 Materials and Methods

### 2.1 Bat Sampling

We collected 2090 blood samples from *E. helvum* individuals across four sites (three wild, one captive) in Ghana from February 2019 to October 2020. Sampling was conducted with ethical clearance from the Ghanaian Council for Scientific and Industrial Research (RPN 001/CSIR‐IACUC/2018), the Zoological Society of London Ethics Committee (ref. IOZ12) and the United States Army Animal Care and Use Review Office (IACUC protocol # CSIR/IRB/AL/VOL1). The four sites consisted of: (1) Accra urban roost, a seasonally‐occupied roost housing up to half a million bats on the grounds of the 37 Military Hospital (sampled five times, *n* = 496 samples); (2) Kumasi urban roost, a resident peri‐urban roost housing up to half a million bats on the grounds of the Kumasi Zoological Garden (sampled four times, *n* = 433 samples); (3) Akosombo rural roost, a roost of unknown population size located in a forested area near the Volta hydroelectric dam (sampled four times, *n* = 386 samples); and (4) a captive colony maintained at the Accra Zoo (sampled five times, *n* = 775 samples, including some resampling of the same 179 individuals). Site coordinates and sampling months are listed in Table S1 and demographic characteristics of each sample are summarised in Table S2.

Wild bats were caught in mist nets while returning to the roost from feeding sites and bats in the captive colony were gathered with landing nets into a large cage. Bats were individually transferred into cotton bags hung from a line until processing. Up to 1 mL of blood (< 1% of body weight, less taken from juveniles based on their size) was collected from the cephalic (propatagial) or brachial vein of each bat and each newly captured bat was microchipped. Age category, sex, weight (in g), forearm length (in mm), female reproductive status (pregnant or lactating) and identifying microchip number were recorded for each individual. Bats were sorted into one of three age categories, based on morphological characteristics: adult (> 24 months), sexually immature (6–24 months), or juvenile (< 6 months) (Peel et al. 2016). For bats in the captive colony, year of birth and numerical age were associated with each individual based on their identifying microchip number. Body condition was calculated as weight/forearm length (following Plowright et al. 2008). All wild bats were released at the site of capture immediately after sample collection.

### 2.2 Serological Assays

Blood samples were screened for paramyxovirus antibodies using an antigen‐based multiplexed microsphere binding assay (Bossart et al. 2007). This method has previously been calibrated for *E. helvum* against Nipah and Hendra virus neutralisation assays (Peel et al. 2013) and used for serological studies in *E. helvum* (Baker et al. 2014; Peel et al. 2018). The production of soluble tetrameric henipavirus attachment (G) glycoprotein antigens has been previously described (Yan et al. 2023). Briefly, serum samples were diluted and incubated with antigen‐coupled microspheres. Antigen–antibody affinity levels were measured via a Luminex FLEXMAP 3D instrument and were reported in median fluorescence intensity (MFI) units. A panel of six paramyxovirus antigens (Table S3) was used in this study: GhV, NiV, HeV, MojV, CedV and MenV, along with a mock bead with no viral antigen.

**FIGURE 1:**
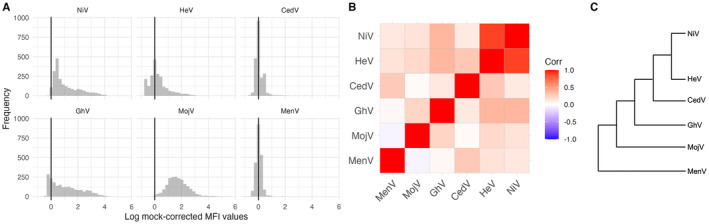
(A) Frequency of log‐MFI values for the six paramyxovirus antigens in the panel. Negative values indicate that the affinity for the antigen is less than the background affinity measured in the absence of viral antigen. (B) Pairwise correlations (calculated with Pearson correlation coefficients) of log‐MFI values across the six paramyxoviruses. (C) Dendrogram indicating relative phylogenetic relationships (not to scale) of viruses included in the Luminex panel (Madera et al. 2022).

All MFI values were log‐transformed (using natural logarithm) prior to analysis to reduce over‐dispersion and then corrected by subtracting the background log‐MFI value for each sample's baseline affinity to mock beads. This correction produces log‐MFI distributions that include some negative values indicating that antigen‐specific affinity is lower than the background affinity (Figure 1A).

### 2.3 Statistical Analyses

All analyses were conducted in R (version 4.2.1; R Core Team 2022) using the mgcv and ggplot2 packages (Wood 2017; Wickham 2016). Correlations between different antigens were assessed with Pearson correlation coefficients and a principal component analysis was performed on the full dataset with all six antigens to visualise the degree of multivariate separation between the sampling sites. We conducted paired Wilcoxon tests to compare each antigen's MFI distribution to the mock distribution and proceeded to run the following analyses for the antigens that differed significantly from the mock distribution. To explore seasonal variation, we ran generalised additive mixed models (GAMMs) of the three wild roosts grouped by Site with Age Category, Sex and Mass/Forearm Length as parametric terms, sampling month as a smoothing term and log‐MFI values as continuous response variables (*n* = 1315).

We then conducted a separate set of analyses on the captive colony data, to further investigate the effect of Age, Sex and Reproductive Status on paramyxovirus seroprevalence in a longitudinal sample with recaptures. Captive adult bats (*n* = 157) were plotted by Sex and GAMMs were run to further quantify temporal patterns by Sex (including Bat ID as a random effect to account for resampling). Rapid increases in antibody binding were also identified within a smaller subset of captive bats measured at each of the five sampling points (*n* = 89), using a conservative cutoff of an increase of 0.978 in log‐MFI value to define such an increase. This cutoff was based on relative increases in log‐MFI values seen in *E. helvum* serum responses to the NiV G‐protein after vaccination with a NiV‐specific vaccine (E. Jax, R. Garnier, G. Tsagkogeorga, V. Warmuth, R. Suu‐Ire, L. Gibson, S. Languon, O. Quaye, S. Rossiter, E. Wright, H. Buczkowski, T. Lambe, S. Gilbert, J. Wood, A. Cunningham, O. Restif, unpublished data). We also ran sensitivity analyses on this log‐MFI increase using two other values (0.5, 1.5). Finally, we examined age‐seroprevalence and reproductive status in the captive colony (*n* = 167 bats) with boxplots of log‐MFI values by age and linear mixed effect models (LMEMs) of the captive bats with Age, Sex, Reproductive Status and Mass/Forearm Length as predictors (with Bat ID included as a random effect to account for resampling of individuals).

In all statistical models, the threshold for statistical significance was adjusted with a Bonferroni correction to account for testing multiple hypotheses on the same dataset with correlated log‐MFI response variables (Figure 1B). Since five models were run (GhV, NiV, HeV, MojV and CedV), the cutoff for significance was set at *p* = 0.01.

## 3 Results

### 3.1 Roost‐Level Patterns, All Sites

Of the six paramyxoviruses included in our panel, four henipaviruses—GhV, NiV, HeV and MojV—displayed the highest proportion of positive log‐MFI values after correction (Figure 1A). A pairwise correlation plot revealed strong cross‐reactivity between NiV and HeV as anticipated and low‐to‐moderate cross‐reactivity between most other pairs of antigens, suggesting that the panel is successfully capturing some of the antigenic diversity in the paramyxoviruses circulating in these colonies (Figure 1B). A principal component analysis of log‐MFI values from the complete dataset revealed overlap between the four roosts, suggesting similar viral antigenic profiles circulating at each site (Figure S1). Even for the antigens with the highest proportion of high values, there was no evidence of bimodal distributions that would enable us to identify an empirical cut‐off for seropositivity (Peel et al. 2013) (Figure 1A).

We focused our subsequent analyses primarily on GhV and NiV, as GhV is the most geographically relevant antigen, while NiV has been used in prior studies as a proxy for henipavirus circulation in this species (Baker et al. 2014; Peel et al. 2018). We report results for other antigens (HeV, MojV and CedV) in Supporting Information. A paired Wilcoxon test revealed that the MenV MFI distribution did not differ significantly from the mock MFI distribution (Table S4) and we therefore excluded this antigen from our analyses.

**FIGURE 2:**
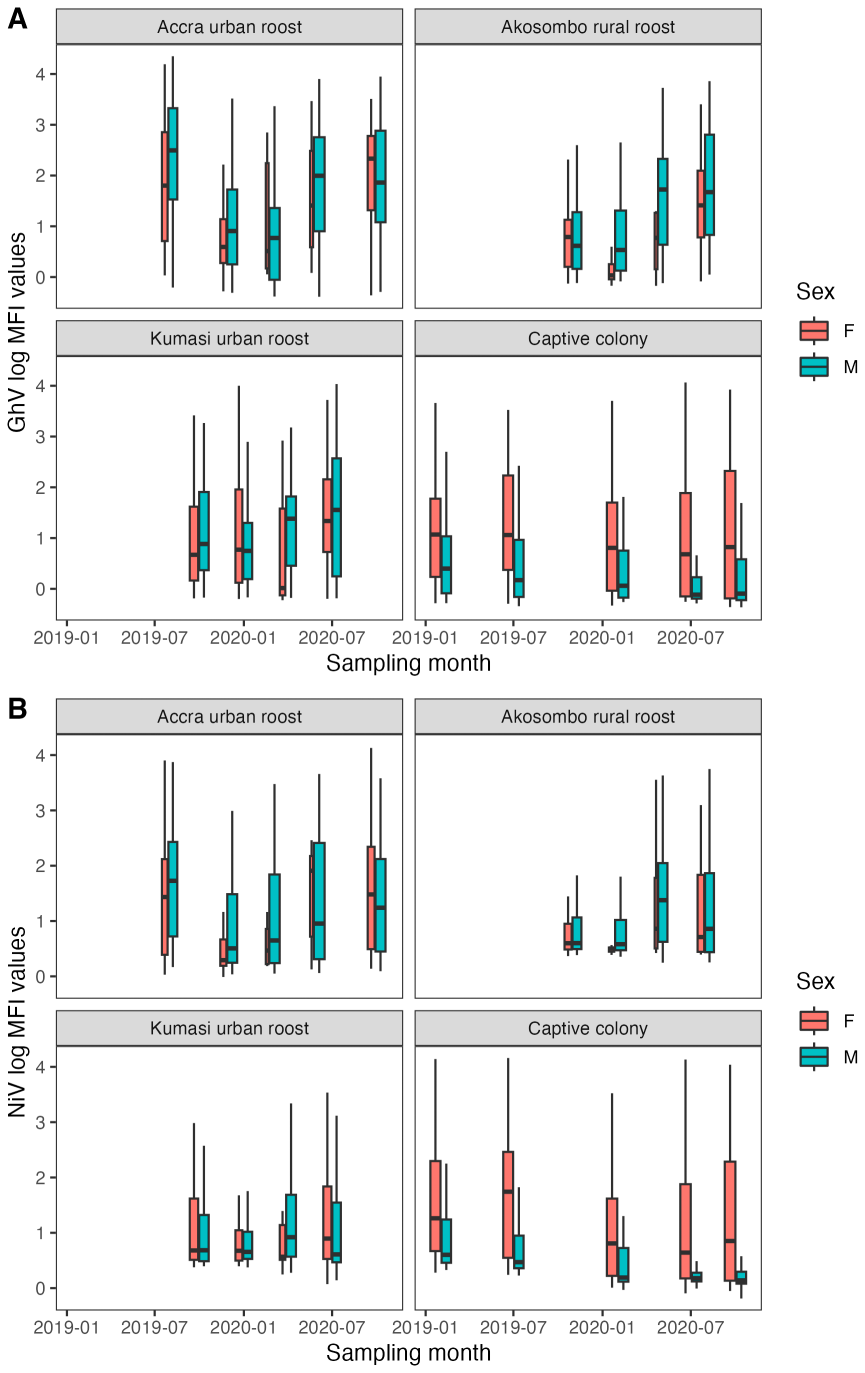
(A) GhV log‐MFI values at each of the four sites across the sampling period, colour coded by sex. (B) NiV log‐MFI values at each of the four sites across the sampling period, colour coded by sex. The width of each box is proportional to the sample size.

GhV and NiV log‐MFI values fluctuated seasonally in all three wild roosts (Figure 2). In these roosts, antibody binding in both sexes peaked around June to August, with the highest values in the Accra urban roost, followed by Akosombo and Kumasi. In the wild roosts, both sexes showed a similar seasonal oscillation. In the captive colony, log‐MFI values were lower for both GhV and NiV. Male antibody binding levels remained relatively flat throughout the sampling period, while females showed the highest annual antibody binding levels in July 2019 and October 2020, corresponding to sampling periods when visibly pregnant female bats were observed in the captive colony (indicating late pregnancy). The seasonal fluctuations in the wild roost antibody binding levels were corroborated by generalised additive mixed models (GAMMs; *n* = 1315) with strong statistical support for a non‐linear dependence on time (*p* < 0.00001 for GhV for all sites and NiV for Accra and Akosombo) (Table 1). There were no statistically significant differences between age categories, sexes, or body conditions for GhV or NiV, except sexually immature bats having GHV log‐MFI values 0.25 units lower than adults (*p* = 0.002).

**TABLE 1:** Smoothing and parametric terms included in generalised additive mixed models (GAMMs) of GhV and NiV log‐MFI values across the three wild roosts and their respective: Effective degrees of freedom (edf), *F* values (*F*) and significance levels (*p*‐value); and estimates, standard errors (SE), *t* statistics (*t* value) and significance levels (*p*r[>*t*]). Results for HeV, MojV and CedV are presented in Table S5.

Antigen					
GhV	**Smoothing term**		**edf**	***F***	***p***
	s(Time):Accra urban roost		3.74	34.76	< 0.00001
	s(Time):Akosombo rural roost		3.55	22.75	< 0.00001
	s(Time):Kumasi urban roost		2.46	12.15	< 0.00001
	**Parametric term**	**Estimate**	**SE**	***t* value**	***p*r(>*t*)**
	Sex (male)	0.15	0.07	2.23	0.0261
	Age (juvenile)	−0.16	0.60	−0.27	0.7883
	Age (sexually immature)	−0.25	0.08	−3.07	0.0022
	Mass/forearm Length	0.02	0.11	0.19	0.8517
NiV	**Smoothing term**		**edf**	***F***	***p***
	s(Time):Accra urban roost		3.55	17.74	< 0.00001
	s(Time):Akosombo rural roost		3.54	7.83	< 0.00001
	s(Time):Kumasi urban roost		1.85	2.87	0.0360
	**Parametric term**	**Estimate**	**SE**	***t* value**	***p*r(>*t*)**
	Sex (male)	0.03	0.06	0.56	0.5725
	Age (juvenile)	0.01	0.51	0.01	0.9900
	Age (sexually immature)	−0.16	0.07	−2.27	0.0232
	Mass/forearm Length	0.08	0.09	0.84	0.3995

### 3.2 Short‐Term Dynamics in the Captive Colony

**FIGURE 3:**
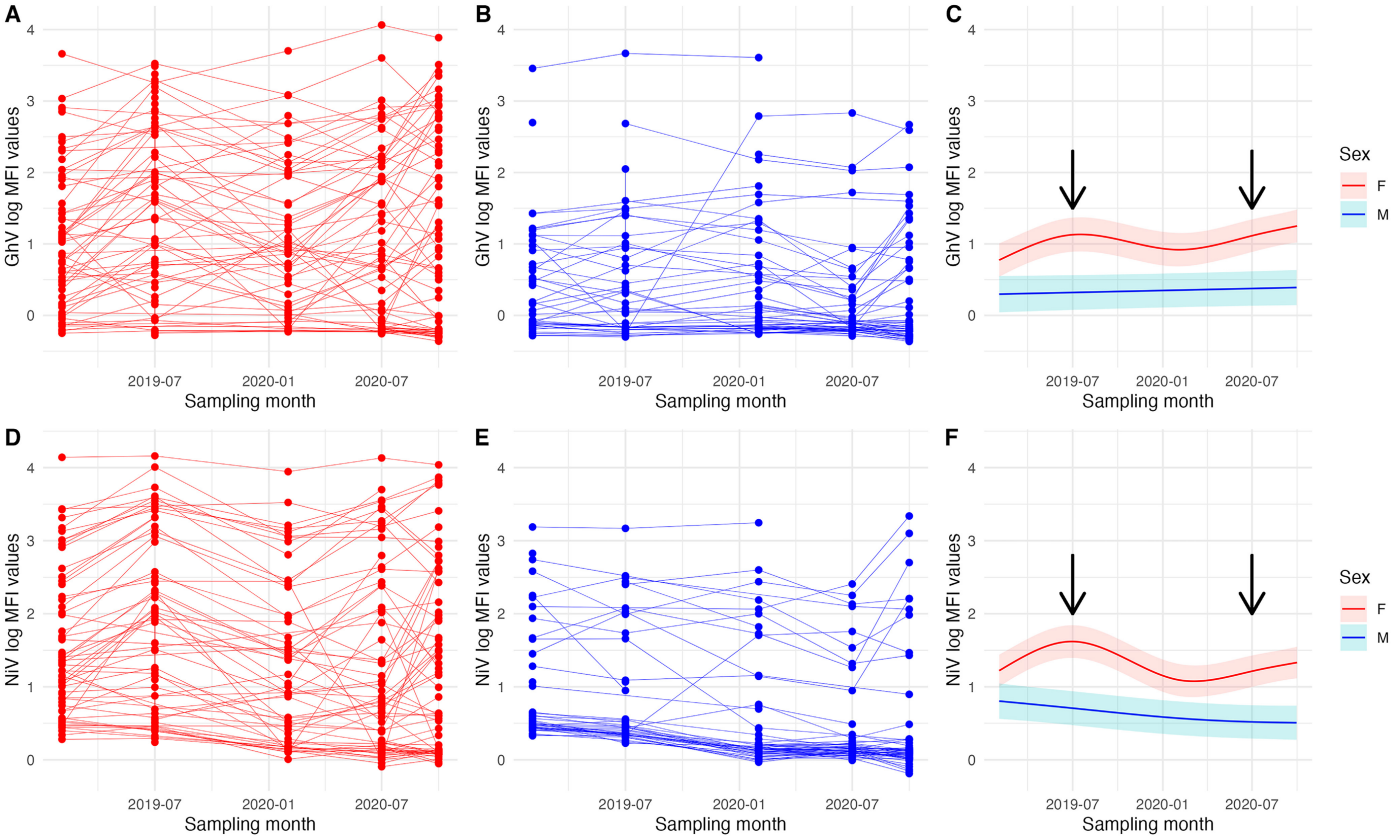
(A) Longitudinal sampling of GhV log‐MFI values from all adult female bats in the captive colony. (B) Longitudinal sampling of GhV log‐MFI values from all adult male bats in the captive colony. (C) Generalised additive mixed models fitted to GhV log‐MFI values from all captive adults, shown with 95% confidence intervals (Table S6). (D) Longitudinal sampling of NiV log‐MFI values from all adult female bats in the captive colony. (E) Longitudinal sampling of NiV log‐MFI values from all adult male bats in the captive colony. (F) Generalised additive mixed models fitted to NiV log‐MFI values from all captive adults, shown with 95% confidence intervals (Table S6). Arrows indicate sampling periods where (likely near‐term) pregnant bats were observed in the colony. Results for HeV, MojV and CedV are presented in Figure S3.

Longitudinal sampling of adult bats (*n* = 157) across five time points in the captive colony reveals that GhV antibody affinity levels in the sera of female bats fluctuated during the sampling period (Figure 3A), while those in all but one male bat remained lower and flatter, oscillating very little (Figure 3B). This corroborates patterns from the captive colony as a whole (Figure 2A). These patterns were further quantified in GAMMs for each sex, where differences in the serological temporal patterns between males and females were statistically significant (Table S6). Female bats' log‐MFI values oscillated during the sampling period, peaking during periods when pregnant bats were observed in the colony, while male bats' antibodies were significantly lower than females' but only showed a slight, non‐significant increase over time (Table S6; Figure 3C).

Similar trends were observed for NiV in the captive colony, with the exception of male antibody binding slightly decreasing over time during the sampling period (Figure 3D–F; Table S6).

We identified 21 and 16 instances of rapid increases in antibody binding (defined as an increase of at least 0.978 in log‐MFI units between two consecutive sampling dates) among the 89 captive bats sampled five times for GhV and NiV, respectively (Table S7). Most rapid increases in both sexes occurred in October 2020 and over 61% (13/21) and 62% (10/16) of all GhV and NiV increases, respectively, were associated with confirmed pregnant or recently pregnant (lactating) female bats. The inclusion of suspected pregnant bats (i.e., females with sudden weight increases consistent with pregnancy) raises these proportions to ~81% of all rapid increases for both antigens. Males accounted for the remaining cases (< 20%) for GhV and NiV during the sampling period. These demographic patterns were generally consistent in sensitivity analyses for all antigens conducted with alternative higher and lower log‐MFI thresholds to define a rapid increase (Table S7).

### 3.3 Long Term Dynamics in the Captive Colony

**FIGURE 4:**
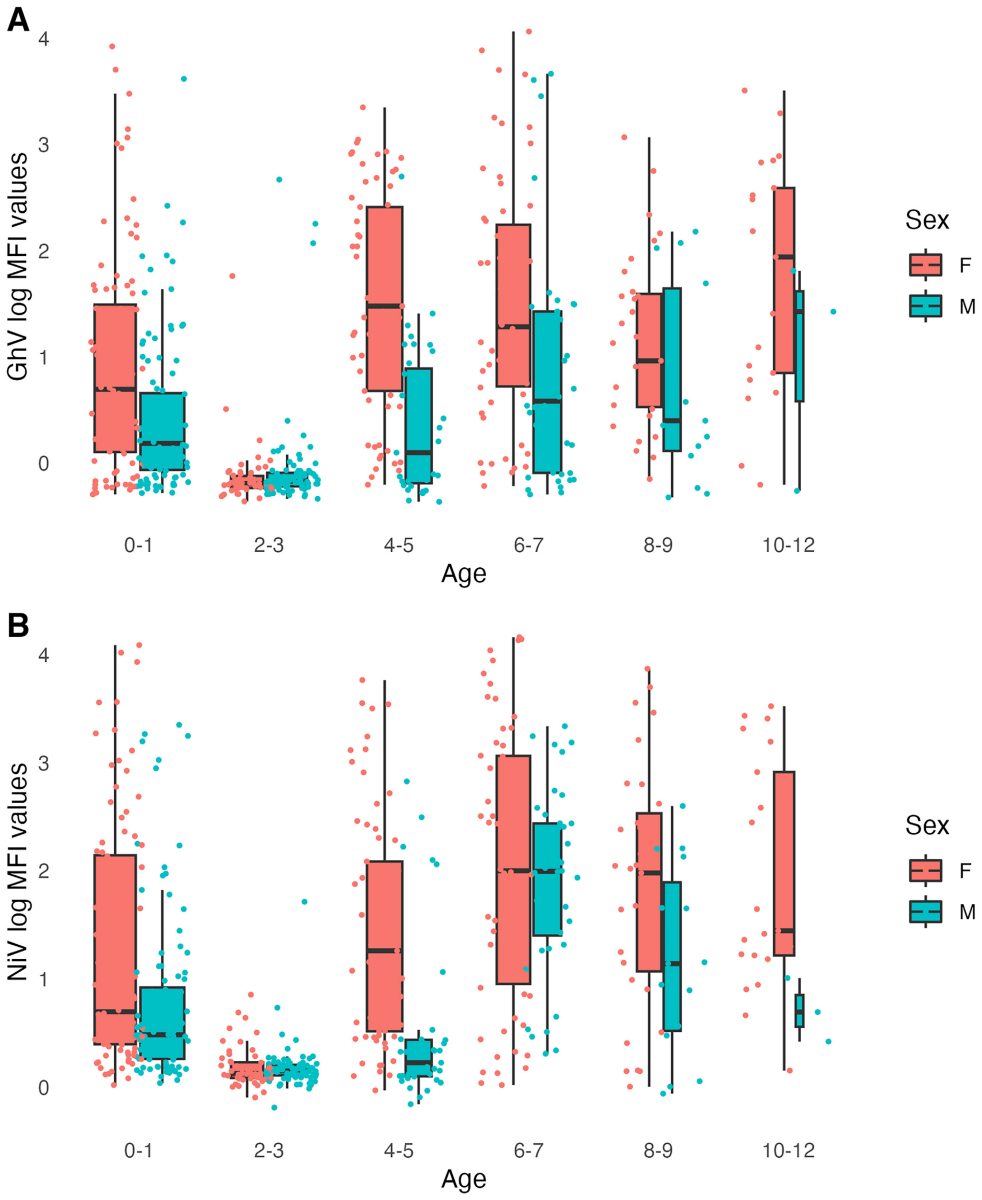
(A) Boxplot of GhV log‐MFI values by age and sex in the captive colony. (B) Boxplot of NiV log‐MFI values by age and sex in the captive colony. The width of each box is proportional to the sample size and jittered points represent individual bats. Results for HeV, MojV and CedV are presented in Figure S4.

MFI distributions by age reveal that for both sexes, GhV and NiV antibodies wane after the 1st year of life, reaching their lowest level by around 2–3 years of age before increasing again at 4–5 years and remaining at similar or higher levels at older ages (Figure 4). This is corroborated by LMEMs (*n* = 167 bats), which suggest that 2–3 year old bats have log‐MFI values 0.72 and 0.82 units lower than 0–1 year old bats, for GhV and NiV, respectively (*p* < 0.00001). In both cases, males had lower log‐MFI values in general than females (0.51 and 0.41 units lower for GhV and NiV, respectively; *p* = 0.0002 and *p* = 0.004) (Table S8). Statistically significant increases in log‐MFI values in later age categories relative to the 0–1 age group (0.62 units higher at age 6–7 for NiV, 0.52 units higher at age 8–12 for GhV) suggest that GhV and NiV antibody affinity levels eventually reach a higher level than the initial maternal antibody titres (Table S8).

## 4 Discussion

Our multifaceted longitudinal dataset spanned 2 years of sampling, six paramyxovirus antigens and four sites (including a closed colony of captive bats), allowing us to examine antibody dynamics across varying spatial and temporal scales. We observed cyclical seasonal fluctuations in GhV and NiV serum antibody levels across all sites, with peaks around July of 2019 and 2020 (Figure 2). The mechanisms driving this pattern in wild roosts are unknown but could be associated with ecological factors such as migration patterns that inflate and shrink *E. helvum* populations at various times of year (Hurme et al. 2022). At the Accra urban roost, for example, hundreds of thousands of bats migrate annually in March/April, leaving a small, largely male resident roost of ~4000 bats from April to October (Peel et al. 2017). This change in population size and structure might contribute to the seasonal increase in antibody binding (Figure 2), for example if most females and young adult bats (both of which tend to have lower antibody affinity levels in the wild roosts; Table 1) have departed. However, our conclusions are limited to the 18‐month sampling period in this study; continued surveillance of these wild roosts over a longer period of time is necessary to confirm cyclical patterns in seroprevalence.

Nipah virus has only been identified in Asia to date and its natural host range is limited to *Pteropus* bats, which are absent from mainland Africa (Eaton et al. 2006). Therefore, NiV antibody binding in this dataset is expected to reflect cross‐reactivity with one or more related but potentially unclassified bat henipaviruses (Drexler et al. 2012; Gibson et al. 2021; Jolma et al. 2021). For all antigens, there are lower log‐MFI values in the captive colony than in the wild roosts (Figure 2; Figure S2), which could be due to lower viral load or diversity at the former site. This may be explained by a bottleneck effect when the founding population was initially closed off a decade prior to this study, or by gradual depletion (akin to genetic drift) of viruses in the small population (Gibson et al. 2021; Jolma et al. 2021). While urine samples from the captive colony, collected during the same period as this study and tested using a henipavirus‐specific PCR (Jolma et al. 2021), contained RNA from at least six distinct “henipa‐like” viruses, we do not have comparable data from any of the wild roosts. There is also a decoupling of male and female antibody binding levels in the captive colony for both GhV and NiV, while the sexes follow the same pattern in wild roosts (Figure 2). This decoupling has occurred over time in the captive colony following isolation from the wild; originally, seroprevalence was similar between sexes in the founding population (Baker et al. 2014).

A closer longitudinal analysis of the captive colony revealed short‐term antibody dynamics related to pregnancy that, in conjunction with lower overall viral load in the captive colony, may explain the aforementioned decoupling between the sexes. For both GhV and NiV, we found dramatic differences in log‐MFI values between the sexes: while adult females experienced oscillations throughout the sampling period, adult males followed steady trends, namely a slight increase in GhV log‐MFI value and decrease in NiV log‐MFI value (Figure 3). The increases in female log‐MFI values coincided with sampling periods where pregnant female bats were observed in the colony. Specifically, over 80% of rapid increases in GhV and NiV antibody binding were recorded in female bats that were either identified as pregnant or lactating or showed statistically significant weight gain consistent with pregnancy (Table S7). These pregnancy‐related patterns are consistent with the dynamics recorded in the founding captive population a decade prior and may be responsible for the apparent peaks in female seroprevalence while male seroprevalence remains flat (Baker et al. 2014).

Our study adds to a growing body of evidence that links reproductive seasonality and henipavirus seroprevalence in fruit bat species (Plowright et al. 2008; Brook et al. 2019). Pregnancy might be associated with higher log‐MFI values for several reasons. The simplest explanation is that mammals may experience immunosuppression during pregnancy and are therefore more susceptible to infection (Lloyd 1983), but this has been challenged in recent decades by more complex evidence suggesting that pregnancy‐induced shifts in the immune system may vary by pathogen (Mor and Cardenas 2010). Alternatively, antibody production might simply be upregulated during pregnancy to increase opportunity for maternal antibody transfer, as has been demonstrated in mice (Bonney 2016). Another possible explanation is that resource reallocations associated with pregnancy could lead to the reactivation of latent infection, which is known to happen with Japanese encephalitis virus in mice (Mathur et al. 1986) and Epstein–Barr virus in humans (Christian et al. 2012). The resurfacing of chronic infection could be due to an increased reliance on humoral immunity during pregnancy, due to the costly metabolic demands of other immune pathways and subsequent downregulation of innate or cell‐mediated immunity (Brook and Dobson 2015). This hypothesised process would have the potential benefit of facilitating maternal antibody transfer to the foetus (through the placenta) or pup (through lactation). Pregnancy‐mediated reactivation of chronic infection in female bats would explain the decoupling of antibody binding levels between the sexes in the captive colony, where overall viral loads are lower and therefore new infections in either sex are rare. Further targeted studies of pregnant bats in the captive colony and in the wild will be necessary to test these hypotheses.

We also found that juveniles (0–1 year old) tend to have higher log‐MFI values (presumably a result of maternally‐derived antibodies) than when re‐tested a year or so later, indicating the waning of maternal antibody concentrations (Figure 4). Increases in log‐MFI values after 2–3 years of age suggest infection and/or reactivation later in life. Taken together with the short‐term dynamics outlined above, we hypothesise that after the initial waning of maternal antibodies, young bats are exposed to viruses resulting in sustained antibody affinity levels, with the addition of low‐amplitude fluctuations associated with pregnancy in females that may be persistently infected.

The seasonal patterns and possible reproductive drivers uncovered here have implications for the prediction and mitigation of viral spillover, which remains understudied for henipaviruses in Africa. In Australia, HeV spillover events often coincide with highly synchronous birth pulses in flying foxes (Plowright et al. 2008). In contrast, the extreme demographic heterogeneity and asynchronous birth pulses of *E. helvum* across the African continent make it difficult and perhaps unwise to extrapolate from the seasonal patterns observed in any one roost (Peel et al. 2017). Nonetheless, our study elucidates the spatial and temporal patterns underlying henipavirus antibody dynamics in *E. helvum* bats in Ghana and represents the first in‐depth analysis of GhV serology, paving the way for the future study of African henipaviruses. *Eidolon helvum* is consumed in West and Central Africa and is the most heavily hunted bat on the continent, creating ample opportunities for contact with humans and subsequent zoonotic viral spillover (Kamins et al. 2011). Indeed, there is evidence of henipavirus seropositivity in individuals who reported butchering *E. helvum* for bushmeat, raising the possibility of undetected spillover events in the past (Pernet et al. 2014). Further research on the drivers of viral circulation in this species has the potential to both improve our understanding of similar bat species and viruses and also inform local public health efforts.

## Author Contributions

**Maya M. Juman:** formal analysis (lead), writing – original draft (lead). **Louise Gibson:** formal analysis (equal), project administration (equal). **Richard D. Suu‐Ire:** conceptualization (equal), investigation (equal). **Sylvester Languon:** investigation (equal). **Osbourne Quaye:** project administration (equal). **Grace Fleischer:** investigation (equal). **Samuel Asumah:** investigation (equal). **E. Rosa Jolma:** investigation (equal), project administration (equal). **Avinita Gautam:** investigation (equal). **Spencer L. Sterling:** investigation (equal). **Lianying Yan:** methodology (equal). **Christopher C. Broder:** resources (equal). **Eric D. Laing:** resources (equal). **James L. N. Wood:** conceptualization (equal), writing – review and editing (equal). **Andrew A. Cunningham:** conceptualization (equal), writing – review and editing (equal). **Olivier Restif:** conceptualization (equal), writing – review and editing (equal).

## Conflicts of Interest

The authors declare no conflicts of interest.

## Statement on Inclusion

Our study brings together scientists from five countries, including the country where the research took place. Ghanaian scientists were involved in designing the study, securing funding and relevant permits, collecting and processing samples and editing the manuscript. The senior Ghanaian and UK‐based investigators have been collaborating since 2008 and have published over 40 papers together.

## Supporting information


Appendix S1.

Data S1.

## Data Availability

Raw data and code are included as Supporting Information and published on GitHub (https://github.com/mayajuman/eidolon‐paramyxos).
