# Supplementary material for: Ecological and Reproductive Cycles Drive Henipavirus Seroprevalence in the African Straw‐Coloured Fruit Bat ( Eidolon helvum )
Source: Ecol Evol. 2024 Nov 11;14(11):e70555. doi: 10.1002/ece3.70555 (PMC11554383; doi:10.1002/ece3.70555)
Supplement: Supplementary file 1 — Appendix S1. [file ECE3-14-e70555-s002.pdf]

Ecological and reproductive cycles drive henipavirus seroprevalence in the African straw-coloured fruit bat (*Eidolon helvum*)

Supplementary Tables S1-8

Supplementary Table S1. Characteristics and locations of sampling sites included in the study.

**Site**	**Type**	**Coordinates**	**Number of bats**	**Sampling months**	**Total *n***
Accra	Wild, urban	5.5882, -0.18239	Up to ~500,000	August 2019, December 2019, March 2020, June 2020, October 2020	496
Kumasi	Wild, peri-urban	6.70002, -1.62493	Up to ~500,000	October 2019, January 2020, April 2020, July 2020	433
Akosombo	Wild, rural	6.171256, 0.064621	Unknown	November 2019, February 2020, May 2020, August 2020	386
Captive colony	Captive	5.625303, -0.203029	~200	February 2019, July 2019, February 2020, July 2020, October 2020	775

Supplementary Table S2. Summary of demographic (sex and age) characteristics of samples from each site, with proportions (as percentages of total sample) presented in parentheses.

Site	Sampling month	Total	Females	Males	Adults	Sexually Immature	Juveniles
Accra (urban)	August 2019	87	25 (29%)	62 (71%)	43 (49%)	41 (47%)	3 (3%)
Accra (urban)	December 2019	106	29 (27%)	77 (73%)	74 (70%)	32 (30%)	0 (0%)
Accra (urban)	March 2020	90	6 (7%)	84 (93%)	74 (82%)	16 (18%)	0 (0%)
Accra (urban)	June 2020	98	7 (7%)	91 (93%)	86 (88%)	12 (12%)	0 (0%)
Accra (urban)	October 2020	115	37 (32%)	78 (68%)	90 (78%)	25 (22%)	0 (0%)
Akosombo (rural)	November 2019	101	39 (39%)	62 (61%)	46 (46%)	55 (54%)	0 (0%)
Akosombo (rural)	February 2020	115	22 (19%)	93 (81%)	81 (70%)	34 (30%)	0 (0%)
Akosombo (rural)	May 2020	74	6 (8%)	68 (92%)	67 (91%)	7 (9%)	0 (0%)
Akosombo (rural)	August 2020	96	34 (35%)	62 (65%)	64 (67%)	32 (33%)	0 (0%)
Kumasi (urban)	October 2019	126	55 (44%)	71 (56%)	70 (56%)	56 (44%)	0 (0%)
Kumasi (urban)	January 2020	100	37 (37%)	63 (63%)	93 (93%)	7 (7%)	0 (0%)
Kumasi (urban)	April 2020	99	24 (24%)	75 (76%)	81 (82%)	18 (18%)	0 (0%)
Kumasi (urban)	July 2020	108	51 (47%)	57 (53%)	40 (37%)	68 (63%)	0 (0%)
Captive colony	February 2019	151	80 (53%)	70 (46%)	103 (68%)	48 (32%)	0 (0%)
Captive colony	July 2019	146	76 (52%)	69 (47%)	104 (71%)	42 (29%)	0 (0%)
Captive colony	February 2020	162	84 (52%)	78 (48%)	114 (70%)	48 (30%)	0 (0%)
Captive colony	July 2020	156	85 (55%)	71 (45%)	121 (78%)	35 (22%)	0 (0%)
Captive colony	October 2020	160	88 (55%)	72 (45%)	124 (78%)	31 (19%)	5 (3%)

Supplementary Table S3. List of viral antigens included in the Luminex panel.

Family	Genus	Virus	Abbreviation
Paramyxoviridae	*Henipavirus*	**Kumasi** virus /*E. helvum*/GHA/2009/GH-M74a	GhV
Paramyxoviridae	*Henipavirus*	**Hendra** virus /*E. caballus*/AUS/1994	HeV
Paramyxoviridae	*Henipavirus*	**Nipah** virus /*H. sapiens*/MYS/2000	NiV
Paramyxoviridae	*Henipavirus*	**Cedar** virus /*Pteropus sp.*/AUS/2012/CG1a	CedV
Paramyxoviridae	*Henipavirus*	**Mojiang** virus /*R. sladeni*/CHN/2014/Tongguan1	MojV
Paramyxoviridae	*Pararubulavirus*	**Menangle** virus /*S. domesticus*/AUS/2001	MenV

Supplementary Table S4. Results of paired Wilcoxon tests comparing each antigen's raw MFI distribution to the background ("mock") distribution.

**Antigen**	**V-statistic**	***p*-value**
GhV	2043561	<0.00001
NiV	2183765	<0.00001
HeV	1529613	<0.00001
MojV	2185060	<0.00001
CedV	1189897	0.00019
MenV	1136206	0.08923

Supplementary Table S5. Smoothing and parametric terms included in generalised additive mixed models (GAMMs) of HeV, MojV, and CedV log-MFI values across the three wild roosts and their respective: effective degrees of freedom (edf), F values (*F*), and significance levels (*p*-value); and estimates, standard errors (*SE*), t statistics (*t* values), and significance levels (*p*r[>*t*]).

**Antigen**					
HeV	**Smoothing term**		**edf**	***F***	***p*-value**
	s(Time):Accra urban roost		3.64	28.96	<0.00001
	s(Time):Akosombo rural roost		3.65	7.04	<0.0001
	s(Time):Kumasi urban roost		1.20	0.58	0.5780
	**Parametric term**	**Estimate**	***SE***	***t* value**	***p*r(>*t*)**
	Sex (Male)	0.02	0.04	0.40	0.6887
	Age (Juvenile)	-0.64	0.36	-1.79	0.0742
	Age (Sexually immature)	-0.10	0.05	-2.00	0.0454
	Mass/Forearm Length	0.05	0.07	0.70	0.4820
MojV	**Smoothing term**		**edf**	***F***	***p*-value**
	s(Time):Accra urban roost		3.78	11.98	<0.00001
	s(Time):Akosombo rural roost		2.28	10.12	<0.0001
	s(Time):Kumasi urban roost		3.75	6.40	0.0005
	**Parametric term**	**Estimate**	***SE***	***t* value**	***p*r(>*t*)**
	Sex (Male)	0.10	0.05	2.14	0.0329
	Age (Juvenile)	-0.15	0.44	-0.35	0.7290
	Age (Sexually immature)	-0.32	0.06	-5.47	<0.00001
	Mass/Forearm Length	-0.30	0.08	-3.68	0.0002
CedV	**Smoothing term**		**edf**	***F***	***p*-value**
	s(Time):Accra urban roost		3.51	42.47	<0.00001
	s(Time):Akosombo rural roost		3.51	4.82	0.0020
	s(Time):Kumasi urban roost		1.91	2.95	0.0335
	**Parametric term**	**Estimate**	***SE***	***t* value**	***p*r(>*t*)**
	Sex (Male)	-0.00	0.02	-0.10	0.9170
	Age (Juvenile)	-0.12	0.16	-0.74	0.4580
	Age (Sexually immature)	0.02	0.02	0.83	0.4100
	Mass/Forearm Length	0.02	0.03	0.85	0.3970

Supplementary Table S6. Smoothing and parametric terms included in generalised additive mixed models (GAMMs) of log-MFI values for GhV, NiV, HeV, MojV, and CedV in captive adult bats, and their respective: effective degrees of freedom (edf), F values (*F*), and significance levels (*p*-value); and estimates, standard errors (*SE*), t statistics (*t* values), and significance levels (*p*r[>*t*]).

**Antigen**					
GhV	**Smoothing term**		**edf**	***F***	***p*-value**
	s(Time):Female		3.67	16.40	<0.00001
	s(Time):Male		1.00	1.38	0.2400
	**Parametric term**	**Estimate**	***SE***	***t* value**	***p*r(>*t*)**
	Sex (Male)	-0.69	0.16	-4.25	<0.00001
NiV	**Smoothing term**		**edf**	***F***	***p*-value**
	s(Time):Female		3.86	20.88	<0.00001
	s(Time):Male		1.86	9.60	0.0001
	**Parametric term**	**Estimate**	***SE***	***t* value**	***p*r(>*t*)**
	Sex (Male)	-0.67	0.15	-4.36	<0.0001
HeV	**Smoothing term**		**edf**	***F***	***p*-value**
	s(Time):Female		3.92	69.17	<0.00001
	s(Time):Male		3.71	75.47	<0.00001
	**Parametric term**	**Estimate**	***SE***	***t* value**	***p*r(>*t*)**
	Sex (Male)	-0.45	0.11	-4.22	<0.0001
MojV	**Smoothing term**		**edf**	***F***	***p*-value**
	s(Time):Female		3.81	42.00	<0.00001
	s(Time):Male		3.32	10.45	<0.00001
	**Parametric term**	**Estimate**	***SE***	***t* value**	***p*r(>*t*)**
	Sex (Male)	-0.14	0.05	-2.84	0.0047
CedV	**Smoothing term**		**edf**	***F***	***p*-value**
	s(Time):Female		3.86	222.10	<0.00001
	s(Time):Male		3.85	161.30	<0.00001
	**Parametric term**	**Estimate**	***SE***	***t* value**	***p*r(>*t*)**
	Sex (Male)	-0.04	0.04	-1.21	0.2266

Supplementary Table S7. Rapid antibody binding increases in a subset of captive bats sampled five times (n = 89). An increase in log-MFI of 0.978 between two sampling points was used to define a rapid increase. Ranges in parentheses indicate estimations using alternative values (1.5 and 0.5, respectively).

**Antigen**		**Feb 2019 to July 2019**	**July 2019 to Feb 2020**	**Feb 2020 to July 2020**	**July 2020 to Oct 2020**	**Total**
GhV	Number of bats showing increases	7 (2-15)	2 (1-7)	2 (1-13)	10 (1-23)	21 (5-58)
	Pregnant or lactating females	6 (2-12)	1 (0-4)	1 (1-9)	5 (1-11)	13 (4-36)
	Males	0 (0-2)	1 (1-3)	0 (0-1)	3 (0-8)	4 (1-14)
NiV	Number of bats showing increases	4 (1-16)	1 (0-1)	3 (1-9)	8 (4-15)	16 (6-41)
	Pregnant or lactating females	4 (1-14)	0 (0-0)	2 (1-6)	4 (2-8)	10 (4-28)
	Males	0 (0-1)	1 (0-1)	0 (0-0)	2 (0-3)	3 (0-5)
HeV	Number of bats showing increases	2 (0-11)	1 (1-1)	3 (0-5)	5 (1-8)	11 (2-25)
	Pregnant or lactating females	2 (0-11)	0 (0-0)	2 (0-3)	2 (1-4)	6 (1-18)
	Males	0 (0-0)	1 (1-1)	0 (0-0)	2 (0-3)	3 (1-3)
MojV	Number of bats showing increases	2 (0-16)	1 (0-3)	0 (0-7)	1 (1-3)	4 (1-29)
	Pregnant or lactating females	2 (0-11)	0 (0-0)	0 (0-5)	0 (0-1)	2 (0-17)
	Males	0 (0-4)	1 (0-2)	0 (0-2)	1 (1-1)	2 (1-9)
CedV	Number of bats showing increases	0 (0-0)	1 (0-2)	0 (0-1)	1 (0-2)	2 (0-5)
	Pregnant or lactating females	0 (0-0)	1 (0-1)	0 (0-1)	0 (0-0)	1 (0-2)
	Males	0 (0-0)	0 (0-1)	0 (0-0)	0 (0-0)	0 (0-1)

Supplementary Table S8. Predictors included in linear mixed-effect models (LMEMs) of log-MFI values for GhV, NiV, HeV, MojV, and CedV in the captive colony, and their respective estimates, standard errors (SE), t statistics (t value), and significance levels (pr[>t]).

**Antigen**	**Predictor**	**Estimate**	***SE***	***t* value**	***pr*(>*t*)**
GhV	Age 2-3	-0.72	0.07	-9.94	<0.00001
	Age 4-5	0.15	0.17	0.86	0.3897
	Age 6-7	0.36	0.16	2.18	0.0304
	Age 8-12	0.52	0.17	3.05	0.0026
	Pregnant	0.15	0.08	1.72	0.0865
	Lactating	0.10	0.14	0.67	0.5046
	Sex (Male)	-0.51	0.14	-3.77	0.0002
	Mass/Forearm Length	-0.09	0.10	-0.87	0.3847
NiV	Age 2-3	-0.82	0.07	-11.37	<0.00001
	Age 4-5	-0.06	0.17	-0.34	0.7362
	Age 6-7	0.62	0.17	3.77	0.0002
	Age 8-12	0.39	0.17	2.25	0.0251
	Pregnant	0.17	0.08	2.09	0.0374
	Lactating	-0.04	0.14	0.32	0.7515
	Sex (Male)	-0.41	0.14	-2.93	0.0039
	Mass/Forearm Length	-0.13	0.10	-1.26	0.2096
HeV	Age 2-3	-1.06	0.06	-17.54	<0.00001
	Age 4-5	-0.24	0.14	-1.70	0.0905
	Age 6-7	0.36	0.14	2.61	0.0098
	Age 8-12	-0.14	0.14	-0.99	0.3244
	Pregnant	0.17	0.07	2.41	0.0165
	Lactating	0.05	0.12	0.45	0.6500
	Sex (Male)	-0.27	0.11	-2.34	0.0205
	Mass/Forearm Length	-0.15	0.09	-1.76	0.0797
MojV	Age 2-3	-0.22	0.05	-4.36	<0.0001
	Age 4-5	0.19	0.07	2.62	0.0095
	Age 6-7	0.28	0.07	4.05	<0.0001
	Age 8-12	0.23	0.07	3.13	0.0020
	Pregnant	0.26	0.06	4.26	<0.0001
	Lactating	0.11	0.10	1.03	0.3060
	Sex (Male)	0.00	0.05	-0.04	0.9649
	Mass/Forearm Length	-0.13	0.07	-1.85	0.0646
CedV	Age 2-3	-0.43	0.03	-12.61	<0.00001
	Age 4-5	-0.01	0.05	-0.16	0.8714
	Age 6-7	0.08	0.05	1.51	0.1319
	Age 8-12	-0.26	0.06	-4.73	<0.00001
	Pregnant	0.00	0.05	0.04	0.9696
	Lactating	-0.04	0.07	-0.56	0.5729
	Sex (Male)	0.01	0.04	0.23	0.8212
	Mass/Forearm Length	-0.03	0.05	-0.71	0.4764

Supplementary Figures S1-4

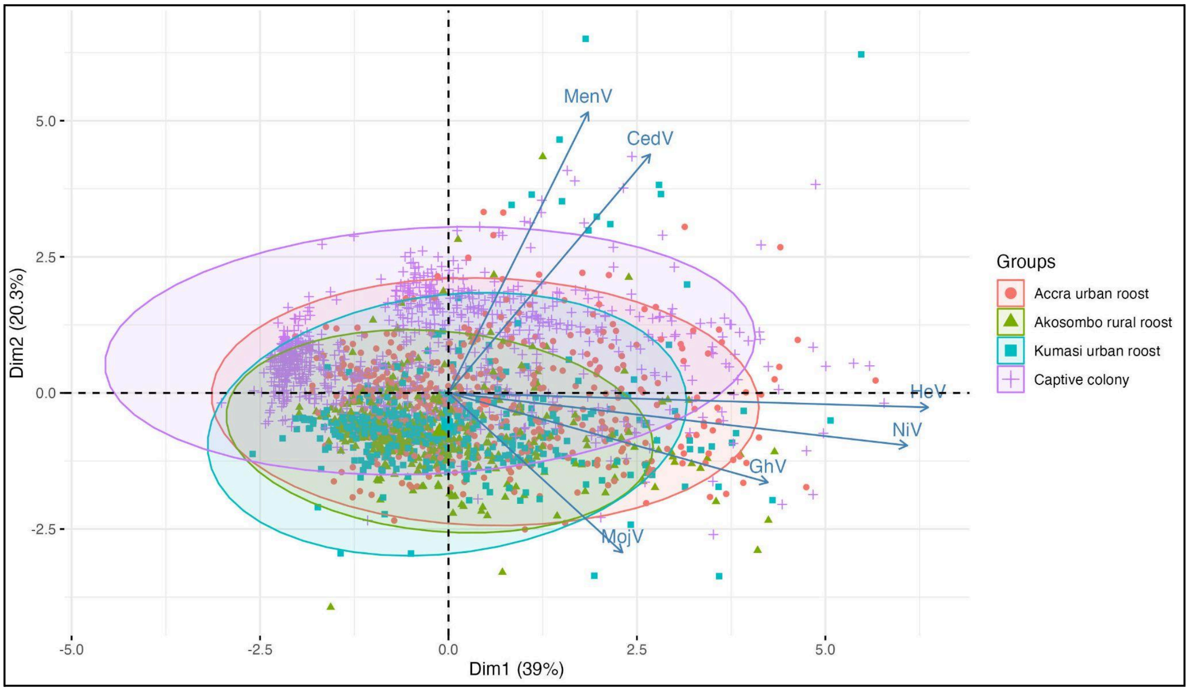

Supplementary Figure S1. Bivariate plot of principal component scores from a principal component analysis of log-MFI values from the complete dataset, colour coded by site.

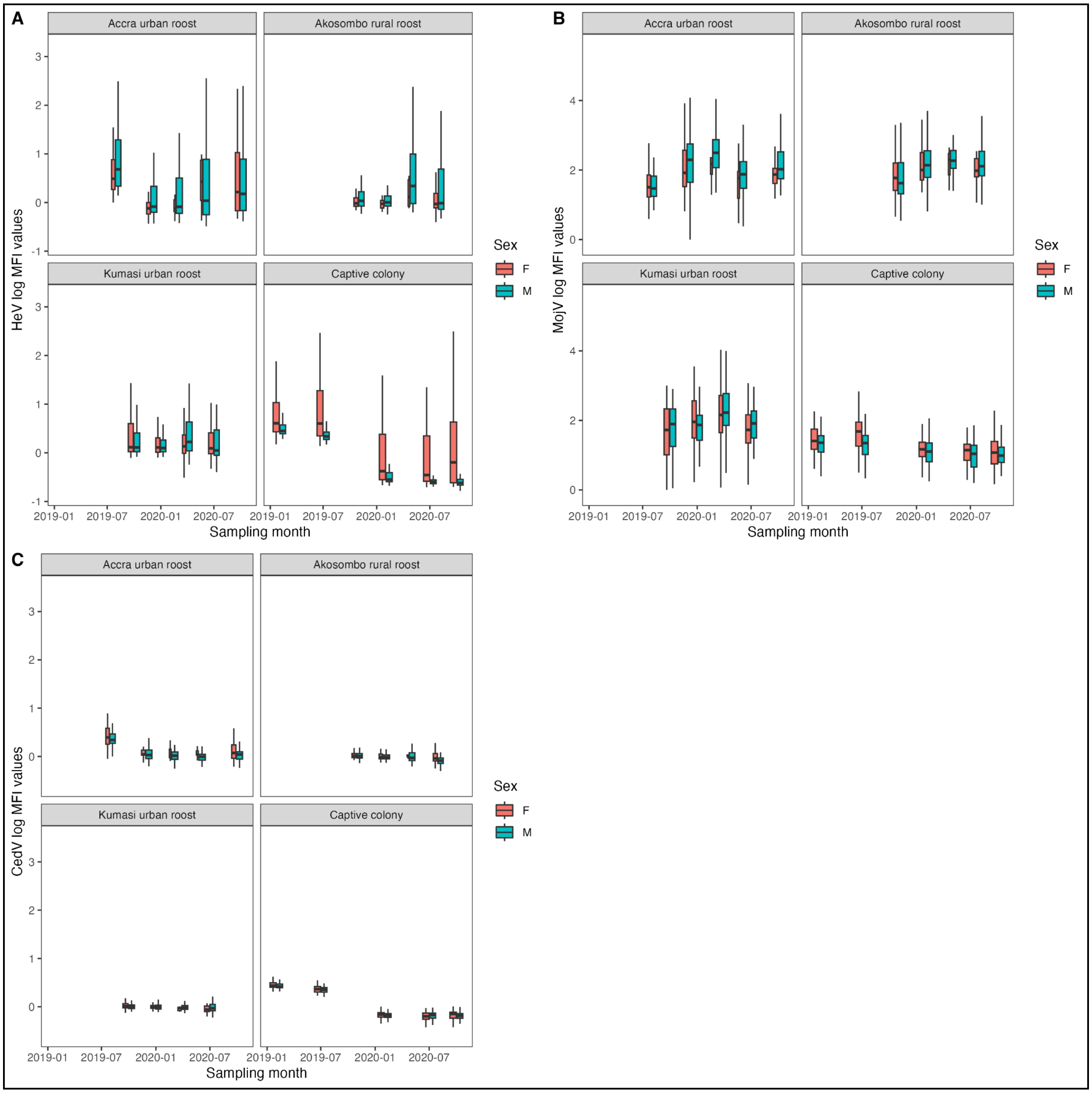

Supplementary Figure S2. A) Boxplot of HeV log-MFI values by sampling month and sex across all sites. B) Boxplot of MojV log-MFI values by sampling month and sex across all sites. C) Boxplot of CedV log-MFI values by sampling month and sex across all sites. The width of each box is proportional to the sample size.

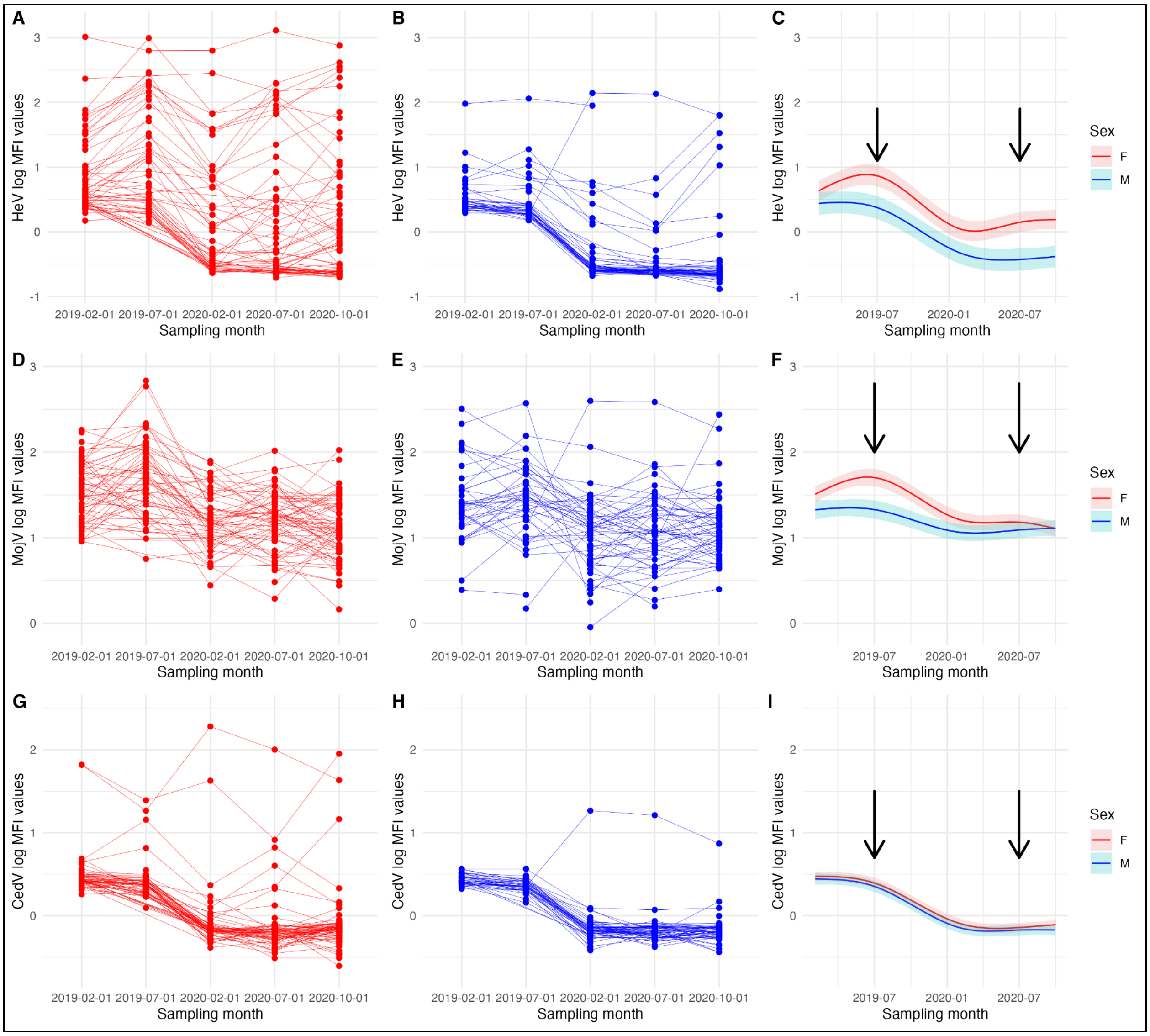

Supplementary Figure S3. A) Longitudinal sampling of HeV log-MFI values from all adult female bats in the captive colony. B) Longitudinal sampling of HeV log-MFI values from all adult male bats in the captive colony. C) Generalised additive mixed models fitted to HeV log-MFI values from all captive adults, shown with 95% confidence intervals (Supplementary Table S6). D) Longitudinal sampling of MojV log-MFI values from all adult female bats in the captive colony. E) Longitudinal sampling of MojV log-MFI values from all adult male bats in the captive colony. F) Generalised additive mixed models fitted to MojV log-MFI values from all captive adults, shown with 95% confidence intervals (Supplementary Table S6). G) Longitudinal sampling of CedV log-MFI values from all adult female bats in the captive colony. H) Longitudinal sampling of CedV log-MFI values from all adult male bats in the captive colony. I) Generalised additive mixed models fitted to CedV log-MFI values from all captive adults, shown with 95% confidence intervals (Supplementary Table S6). Arrows indicate sampling periods where (likely near-term) pregnant bats were observed in the colony.

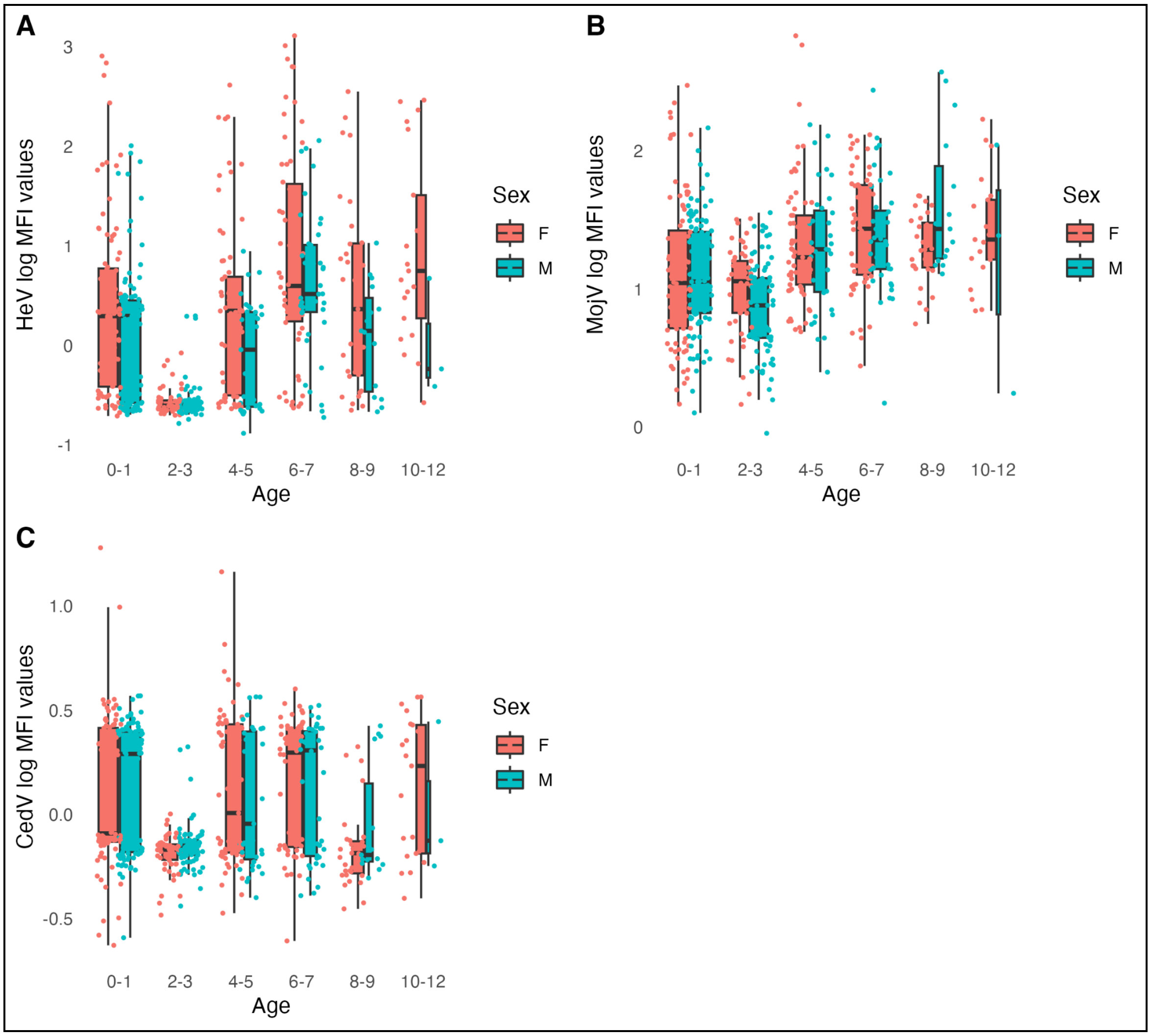

Supplementary Figure S4. A) Boxplot of HeV log-MFI values by age and sex in the captive colony. B) Boxplot of MojV log-MFI values by age and sex in the captive colony. C) Boxplot of CedV log-MFI values by age and sex in the captive colony. In all boxplots, the width of boxes represents the relative sample size in each age/sex category and jittered points represent individual bats.
